# Answer to “Dental Miscellaines”

**Published:** 1872-10

**Authors:** W. E. Driscoll


### Answer to "Dental Miscellaines."

BY W. E. DRISCOLL.

After a few months rest the writer, under the head of "Dental Miscellaines", in the DENTAL REGISTER for August, 1872 returns to the attack on oxychloride of zinc as a capping for exposed dental pulps. This gentleman's views I take to be erroneous, and as a consequence will do harm if not persistently combatted every time they are brought to public notice This I consider has been well done heretofore, but those who have taken the trouble to do this in the past may tire of what may seem an endless task, and for a time allow him to claim that his side has triumphed, that exposed pulps can not be saved, thus leading many farther astray than months of earnest truthful teaching will be likely to counteract and correct.

That we will never have anything better than oxychloride of zinc for covering exposed pulps I do not affirm. I hope we may. For immediate contact with the pulp a solution of gutta percha, or collodion may be better, but of this we are by no means certain as some of those who use these materials between the cement and the pulp complain of considerable disappointment and failure.

Our unbelieving friend wants to know if any have "found bony arches thrown over exposed pulps where the os-artificial had been previously used." I have not for the very good reason that all are doing so well that they can not be spared even to gratify so laudable a curiosity as that of our friend.

He is against the use of oxychloride of zinc "in very young or immature teeth." What then will he do with these teeth? Extract them at this the very age, that of youth when they are most desired of any age?

Does he not know that when "young or immature" they are, as compared with old and denser teeth, unfit for root filling? How is the process of full development to be carried out in immature pulpless teeth? Instead of not using it in "young or immature" teeth, it is the only resort for saving them that I know of, or that he does as it would seem by the report of his "Case No. 1," in which he filled with gold and then extracted the tooth in a few weeks afterward.

How utterly perverse and absurd this seems to one constantly in the habit of saving exposed pulps. And since the tooth could have been saved so easily in this "Case No. 1," the boy should have been paid for the damage done him if such a loss could be estimated or repaid in dollars and cents.

He says, "The ossification of the teeth is from the rudimental pulp, and when once completed is done forever." Now what student of dentistry of one year's experience does not know that in cases of abrasion of the cutting edges of the teeth in elderly persons where it extends below the former limit of the pulp that a new covering of dentine is thrown out for the protection of the pulp? This is very common and yet our writer says it never happens. But the errors of these "Dental Miscellanies" are too numerous to be noticed in detail, and hoping no one will be deterred from learning to save pulps by what he has written.

